# Genome-wide CRISPR/Cas9 screening identifies host factors critical for antiviral defense against equine herpesvirus type 1

**DOI:** 10.3389/fimmu.2026.1764863

**Published:** 2026-02-02

**Authors:** Ziqian Li, Lijuan Ge, Tingting Yu, Shubing Lv, Qiang Fu, Huijun Shi

**Affiliations:** 1 College of Veterinary Medicine, Xinjiang Agricultural University, Urumqi, Xinjiang Uygur Autonomous Region, Urumqi, China; 2 College of Veterinary Medicine, Xinjiang Agricultural University Key Laboratory of New Drug Research and Innovation for Herbivorous Animals of Xinjiang, Urumqi, Xinjiang Uygur Autonomous Region, Urumqi, China

**Keywords:** CRISPR/Cas9, EHV-1, library screening, viral host factors, viral replication

## Abstract

**Introduction:**

Equine herpesvirus type 1 (EHV-1) is a major veterinary pathogen causing significant economic losses in the livestock industry. Despite its impact, effective vaccines and targeted antiviral strategies remain limited, largely due to an incomplete understanding of host factors regulating viral replication and pathogenesis.

**Methods:**

To systematically identify host genes essential for EHV-1 infection, we established a BHK-21 cell line stably expressing Cas9 and performed a genome-wide CRISPR/Cas9 knockout screen using a pooled lentiviral single-guide RNA library. Significantly enriched candidate genes from positive selection were validated by generating knockout cell lines. Viral replication and protein expression were assessed using quantitative polymerase chain reaction and Western blot analysis. Pathway enrichment and protein interaction network analyses were subsequently conducted.

**Results:**

Genome-wide CRISPR/Cas9 screening identified multiple host factors critical for EHV-1 replication. Pathway enrichment analysis revealed that these genes were involved in key cellular signaling and regulatory networks associated with viral infection. Functional validation demonstrated that knockout of selected host genes significantly suppressed EHV-1 replication and viral protein synthesis.

**Discussion:**

These findings highlight essential host determinants required for EHV-1 replication and suggest that targeting host factors may represent a promising strategy for antiviral intervention. This study provides a foundation for the development of host-directed immunotherapeutic and antiviral approaches against EHV-1 infection.

## 1 Introduction

Equine herpesvirus type 1 (EHV-1) is a member of the subfamily Alphaherpesvirinae within the Herpesviridae family. It is a linear double-stranded DNA virus with a genome of approximately 150 kb that encodes more than 70 open reading frames (1). Following EHV-1 infection in equines, the initial clinical signs typically include fever, nasal discharge, and respiratory symptoms. Subsequently, the infection may progress to more severe manifestations, such as abortion, fetal weakness, and neurological disease. The most common and destructive outcome is equine herpesvirus encephalomyelitis. The mortality rate in neurological cases can reach 30%–50% (2, 3), resulting in substantial economic losses to the horse industry.

Although existing vaccines and antiviral agents can limit viral spread to some extent, EHV-1 outbreaks continue to be frequently reported in clinical practice. Notably, many viruses have evolved multiple immune evasion strategies. Using structural biology and neutralization assays, Krummenacher et al. demonstrated that envelope glycoprotein gC of herpes simplex virus type 1 (HSV-1) shields the gD epitope through steric hindrance, thereby protecting viral particles from neutralizing antibodies. In addition, co-immunoprecipitation and endocytosis pathway-tracking studies by Lin et al. showed that the pUL56 protein of EHV-1 promotes the degradation of major histocompatibility complex class I molecules through a dynein-dependent endocytic pathway, thereby inhibiting CD8^+^ T-cell antigen presentation (4, 5). Virus-encoded immunomodulatory proteins play a critical role in pathogenesis; however, viral replication also depends heavily on multiple host cell molecular mechanisms (6, 7). Therefore, systematically elucidating virus–host interaction mechanisms from the perspective of host factors has become an important direction in virology and antiviral therapy research.

In recent years, rapid advances in gene-editing technologies—particularly the widespread application of the CRISPR/Cas9 system for gene knockout, knock-in, and genome-wide screening—have enabled systematic identification of host factors on a genome-wide scale (8, 9). CRISPR/Cas9-mediated genome-wide knockout screening allows thousands of host genes to be disrupted simultaneously within a single experimental system through the construction of large-scale single-guide RNA (sgRNA) libraries covering the human genome. This approach enables direct assessment of the effects of individual host genes on viral infection, replication, and transmission. Such high-throughput screening strategies provide a powerful technical framework for identifying key host factors involved in the viral life cycle (10, 11).

On the basis of accumulating evidence, genome-wide CRISPR/Cas9 screening has become an indispensable tool for systematically uncovering host factors that mediate viral invasion, replication, and dissemination (10). For example, Suzuki et al. used genome-wide CRISPR/Cas9 screening to identify host factors required for HSV-1 infection, demonstrating that PAPSS1, a key enzyme in heparan sulfate biosynthesis, is essential for HSV-1 adsorption and entry, with its knockout markedly reducing viral infection efficiency (12). Similarly, Han et al. employed a human whole-genome CRISPR/Cas9 knockout library to identify the sialic acid transporter SLC35A1 as a core host factor required for influenza virus entry in A549-Cas9 cells and further revealed the involvement of multiple host genes regulating innate immune responses in viral replication (13). In addition, Shue et al. identified RACK1 as a critical host factor for flavivirus replication through genome-wide CRISPR/Cas9 knockout screening, showing that loss of RACK1 significantly impairs viral RNA replication and suppresses viral proliferation (14). Taken together, these studies demonstrate that CRISPR-based screening has become a central approach for systematically identifying key host factors across diverse viral infections and plays an increasingly important role in elucidating the molecular mechanisms underlying viral infection and host regulation (11).

Under *in vitro* conditions, baby hamster kidney fibroblast-21 (BHK-21) cells have been widely used to investigate EHV-1 infection mechanisms and virus–host interactions because of their high susceptibility to EHV-1 and their ability to support efficient viral replication (15–17). Consequently, they have become an important cellular model for screening host factor functions. Therefore, this study first established a stable monoclonal BHK-21 cell line expressing the Cas9 protein. Using this cell line, a genome-wide CRISPR/Cas9 screen was performed to identify host genes that play critical roles in EHV-1 infection. Based on sequencing data, bidirectional host factors regulating EHV-1 replication were further analyzed, and Kyoto Encyclopedia of Genes and Genomes and Gene Ontology functional enrichment analyses were conducted on the top 10 positively correlated genes. In addition, the effects of knocking out positively acting host genes on EHV-1 replication levels and virus-induced cytopathic effects (CPEs) were evaluated. Disruption of key host factors significantly reduced EHV-1 replication and attenuated virus-induced cytopathic changes. These findings indicate that modulation of host gene expression to regulate viral replication offers novel insights into potential therapeutic targets for EHV-1.

## 2 Materials and methods

### 2.1 Plasmids, cells, and viruses

The plasmids used for lentiviral packaging—SPAX2 (12260), pMD2.G (12259), and the Mouse Two-Plasmid Activity-Optimized CRISPR Knockout Library (1000000053)—were purchased from Addgene (USA). Endura^™^ ElectroCompetent Cells were obtained from Lucigen, Inc. (USA). The plasmids LentiCRISPRv2-Puro-Plekhg5, LentiCRISPRv2-Puro-Alox15, LentiCRISPRv2-Puro-Slc35a2, LentiCRISPRv2-Puro-Olfr1090, LentiCRISPRv2-Puro-Psmc2, LentiCRISPRv2-Puro-Arhgef38, LentiCRISPRv2-Puro-Gpr56, LentiCRISPRv2-Puro-Gm14548, LentiCRISPRv2-Puro-Mrpl54, and LentiCRISPRv2-Puro-Sdk1 were constructed by Beijing Qingke Biotechnology Co., Ltd. (China).

Human embryonic kidney 293T (HEK293T), BHK-21, BHK-21-Cas9 cells, and the EHV-1 XJ2015 strain were maintained at the College of Veterinary Medicine, Xinjiang Agricultural University. Cells were cultured in Dulbecco’s modified Eagle medium (06-1055-57-1) supplemented with 10% fetal bovine serum (Kibbutz Beit Haemek, Israel; 04-001-1), 100 U/mL of penicillin, and 100 U/mL of streptomycin (Shanghai Biyuntian Biotechnology Co., Ltd., China) at 37°C in a humidified atmosphere containing 5% CO_2_.

When cell confluence reached approximately 70%, cells were infected with EHV-1 at a multiplicity of infection (MOI) of 0.5. After incubation for 2 h at 37°C with 5% CO_2_, the supernatant was removed, and the cells were washed three times with phosphate-buffered saline. Fresh Dulbecco’s modified Eagle medium containing 2% fetal bovine serum was then added, and the cells were returned to the incubator. When the CPE reached approximately 70%, the viral supernatant was harvested, centrifuged at 12,000 rpm for 10 min at 4°C, filtered through a 0.45-µm microporous membrane, aliquoted, and stored at −80°C for subsequent use.

### 2.2 Preparation of genome-scale CRISPR knockout library lentivirus

HEK293T cells were seeded in culture flasks. When cell confluence reached approximately 80%, cells were co-transfected with the Mouse Two-Plasmid Activity-Optimized CRISPR Knockout Library plasmid and the control plasmid pLenti-GFP (green fluorescent protein), together with the packaging plasmids pMD2.G and pSPAX2, at the indicated ratios. The culture medium was replaced with fresh medium 3 h after transfection. Supernatants were collected at 48 h post-transfection and filtered through a 0.45-µm microporous membrane to remove cell debris, yielding the genome-scale CRISPR knockout (GeCKO) library lentivirus and the GFP-positive control lentivirus. Lentivirus production efficiency was assessed by evaluating GFP expression in the pLenti-GFP control group under a fluorescence microscope. The lentiviral stocks were then aliquoted and stored at −80°C until further use.

### 2.3 GeCKO library lentivirus titration and transduction

BHK-21-Cas9 monoclonal cells (1 × 10^5^) were seeded into six-well plates, and serial dilutions of the GeCKO library lentivirus were added to determine optimal transduction conditions. Blank control wells received no lentivirus. Cells were cultured at 37°C in a humidified atmosphere containing 5% CO_2_, and the medium supplemented with puromycin (4 µg/mL) was added for 3 days of selection. Surviving cells were subsequently collected and counted.

To ensure that most cells harbored a single sgRNA and that gene perturbations remained independent, library transduction was performed at an MOI <0.3. To maintain sufficient library coverage, defined as at least 500 cells per sgRNA, 2.17 × 10^8^ BHK-21-Cas9 cells were infected at an MOI of 0.3. After 48 h, puromycin selection was applied for an additional round of screening. Complete death of non-transduced wild-type BHK-21 cells confirmed effective selection pressure. Subsequently, 6.5 × 10^7^ surviving cells were collected as the non-infected control group and stored at −80°C for downstream sequencing and analysis.

### 2.4 Whole-genome CRISPR/Cas9 screening

EHV-1 was propagated at scale, and viral titers were determined. BHK-21 cell lines stably expressing Cas9 were established and infected with EHV-1 at different MOIs. The viral dose that resulted in complete cell death by day 7 post-infection was determined and used for subsequent screening experiments. Surviving cells from the experimental group were collected 7 days after infection at the designated MOI and stored at −80°C.

Genomic DNA was extracted from both control and experimental groups using the Zymo Research Quick-gDNA Midiprep Kit, and DNA concentrations were quantified. sgRNA target regions were amplified by two rounds of polymerase chain reaction (PCR). In the first round, genomic DNA was used as the template with NEBNext High-Fidelity PCR Master Mix and primers NGS-indel-R1-Fwd and NGS-indel-R1-Rev. The resulting products were then used as templates for the second round of PCR with NEBNext High-Fidelity PCR Master Mix and primers NGS-indel-R2-Fwd and NGS-indel-R2-Rev. PCR products from the same sample were pooled and purified using the QIAquick Gel Extraction Kit, followed by high-throughput sequencing on the Illumina HiSeq platform performed by Geneviz Biotechnology Co., Ltd, Suzhou, China.

sgRNA enrichment or depletion was determined by comparing relative sgRNA abundance levels before and after screening. High-throughput CRISPR/Cas9 sequencing data were analyzed using MAGeCK-VISPR and MAGeCK-NEST to perform positive and negative selection analyses while controlling the false discovery rate. Gene-level significance was assessed by evaluating the concordant enrichment or depletion of multiple sgRNAs targeting the same gene, thereby identifying host factors essential for EHV-1 infection and replication.

To assess cross-species compatibility of the mouse GeCKO sgRNA library in BHK-21 cells, all sgRNA sequences were computationally aligned to the *Mesocricetus auratus* reference genome. Among all sgRNAs, 94.2% showed perfect matches to the hamster genome, whereas 5.8% failed to align. sgRNAs with perfect or near-perfect matches within coding regions were retained for downstream analysis, whereas poorly aligned sgRNAs were excluded. Gene-level enrichment was considered valid only when multiple sgRNAs targeting conserved regions exhibited consistent enrichment or depletion patterns.

### 2.5 Establishment of CRISPR/Cas9 knockout cell lines

The plasmids LentiCRISPRv2-Puro-Plekhg5, LentiCRISPRv2-Puro-Alox15, LentiCRISPRv2-Puro-Slc35a2, LentiCRISPRv2-Puro-Olfr1090, LentiCRISPRv2-Puro-Psmc2, LentiCRISPRv2-Puro-Arhgef38, LentiCRISPRv2-Puro-Gpr56, LentiCRISPRv2-Puro-Gm14548, LentiCRISPRv2-Puro-Mrpl54, and LentiCRISPRv2-Puro-Sdk1 were co-transfected into HEK293T cells together with the packaging plasmids pMD2.G and pSPAX2. BHK-21 cells were seeded into six-well plates and cultured until approximately 80% confluence. Lentiviral particles carrying individual knockout constructs were then used to transduce BHK-21 cells, and successfully transduced cells were selected using puromycin. Gene knockout efficiency was validated by quantitative PCR (**Table 1**; **Supplementary Figure S1**).

**Table 1:** Primers for qPCR.

Gene name	Forward sequence	Reverse sequence
*Plekhg5*	ACATCTAGACAAATATGGTGTGTCA	CACAGTGCTGTGGAACTTGC
*Alox15*	GAGTCGTCACTCTCCAAGTGTT	GAATTCTGCCTCCGAGTTCC
*Slc35a2*	CTTTCCAGCCTTCCCCGAG	CCTTGGTGAGCAACTTTGGC
*Olfr1090*	TCTGAATTGCAGGCCCCATT	GTACATGGGTGTTTGCAGGC
*Psmc2*	GTGCAGAGGACCATGTTGGA	AGATGTGAGTCCGACCCTCT
*Arhgef38*	CCTTCAGCACATAGAGGCCAT	CCGCAGTTCTCCTCTCCATC
*Gpr56*	CACCGGAAGCTAGAAGGGTAG	CCCAGAAGTCACCACCTCTGTC
*Gm14548*	AGCAACCCCTGCGACTAAAA	CCTGGTTCTCTGTGGCTGAG
*Mrpl54*	GTACCCCGAGTGGCTATTCC	CGCCAGTACTCTCGGGATTC
*Sdk1*	CGCTGGCGCAAGATGATG	GAGCTCACTGTCGTTGTGGA
*EHV-1*	GCCATACGTCCCTGTCCGACAA	CCTCCACCTCCTCGACGATGC

### 2.6 Cell viability assay

Wild-type BHK-21 cells and scramble control cells, as well as *Plekhg5*, *Alox15*, *Slc35a2*, *Olfr1090*, *Psmc2*, *Arhgef38*, *Gpr56*, *Gm14548*, *Mrpl54*, and *Sdk1* knockout BHK-21 cells, were seeded into 96-well plates at a density of 5 × 10^3^ cells per well, with eight replicates per group. Cells were cultured in 100 µL of medium per well for 48 h. Subsequently, a Cell Counting Kit-8 reagent was added to each well, followed by incubation for 1.5 h. Absorbance was measured at 450 nm (OD_450_) using a microplate reader. Relative cell viability was calculated as follows: Relative cell viability (%) = (OD_450_ of experimental group/OD_450_ of control group) × 100%.

### 2.7 Virus titration

Collected virus samples were serially diluted 10-fold in Dulbecco’s modified Eagle medium, ranging from 10^−1^ to 10^−10^, and inoculated onto monolayers of BHK-21 cells cultured in 96-well plates. Prior to inoculation, cells were washed three times with phosphate-buffered saline. For each dilution, 100 µL of virus suspension was added per well, with five replicate wells for each dilution. Plates were incubated at 37°C in a humidified atmosphere containing 5% CO_2_ for 3–5 days, and CPEs were monitored and recorded daily.

Viral titers were calculated using the Reed–Muench method and expressed as the 50% tissue culture infectious dose per milliliter. Each experiment was performed using independent virus preparations, and titers were determined based on the proportion of CPE-positive wells at each dilution.

### 2.8 Real-time quantitative PCR

Knockout cells and scramble control cells in the logarithmic growth phase were seeded as required. When cell confluence reached approximately 70%, cells were infected with EHV-1 at an MOI of 0.1. Infected cells were harvested at 0, 6, 12, 24, 36, and 48 h post-infection. Cells were washed three times with phosphate-buffered saline (PBS), and total cellular DNA was extracted from both groups using the Quick-gDNA™ Midiprep Kit (Zymo Research, USA). DNA concentration was measured and normalized to 100 ng per sample.

Quantitative real-time PCR (qPCR) was performed using a QuantStudio™ 6 Flex Real-Time PCR System (Applied Biosystems, USA) to quantify EHV-1 DNA levels. The EHV-1-specific primers were designed and validated during the establishment of a laboratory-developed EHV-1 qPCR detection assay, targeting a conserved region of the viral genome (GenBank accession no. M36298; nucleotide positions 1207–1509 bp). The primer sequences were as follows: forward primer, 5′-GCCATACGTCCCTGTCCGACAA-3′; reverse primer, 5′-CCTCCACCTCCTCGACGATGC-3′.

### 2.9 Western blot analysis

Cells were lysed on ice for 45 min using protein lysis buffer (50 mM of Tris–HCl, pH 7.4, 150 mM of NaCl, 1% Triton X-100) supplemented with protease inhibitors. Lysates were centrifuged at 14,00 *g* for 15 min at 4°C to remove cell debris, and the supernatants were collected. Protein samples were mixed with 6× Laemmli buffer (0.375 M of Tris–HCl, 9% sodium dodecyl sulfate, 50% glycerol, 0.03% bromophenol blue, 9% β-mercaptoethanol) and denatured by boiling at 95°C for 10 min.

Denatured proteins were separated by 10% sodium dodecyl sulfate–polyacrylamide gel electrophoresis and transferred onto polyvinylidene difluoride membranes (Merck Millipore, Germany). Membranes were blocked for 1 h in either phosphate-buffered saline containing 0.05% Tween-20 and 5% skim milk powder or Tris-buffered saline containing 0.01% Tween-20 and 5% bovine serum albumin. After blocking, membranes were incubated overnight at 4°C with EHV-1-positive serum (1:200 dilution) and an anti-glyceraldehyde 3-phosphate dehydrogenase antibody (10494-1-AP; 1:5,000 dilution). Membranes were then washed three times with Tris-buffered saline containing Tween-20 and incubated with horseradish peroxidase-conjugated secondary antibodies (1:5,000 dilution) at room temperature for 1 h, followed by three additional washes.

Protein signals were detected using the ECL Plus Western Blotting Detection System (Pierce, GE Healthcare, Little Chalfont, UK) and captured using a chemiluminescence imaging system (Intas Science Imaging, Germany). Band intensities were quantified using ImageJ software.

### 2.10 Detection of CPEs caused by EHV-1

Cells were infected with EHV-1 at a uniform MOI of 0.5. CPEs were observed under a microscope at 12, 24, 36, and 48 h post-infection.

### 2.11 Statistical analysis

Statistical analyses were performed using SPSS version 25.0 and GraphPad Prism version 8.0. Differences between groups were assessed using independent-samples *t*-tests or one-way analysis of variance, as appropriate. Data are presented as the mean ± standard error of the mean. Statistical significance was defined as *P < 0.05, **P < 0.01, and ***P < 0.001.

## 3 Results

### 3.1 Establishment and characterization of a BHK-21 monoclonal cell line stably expressing Cas9

**Figure 1:**
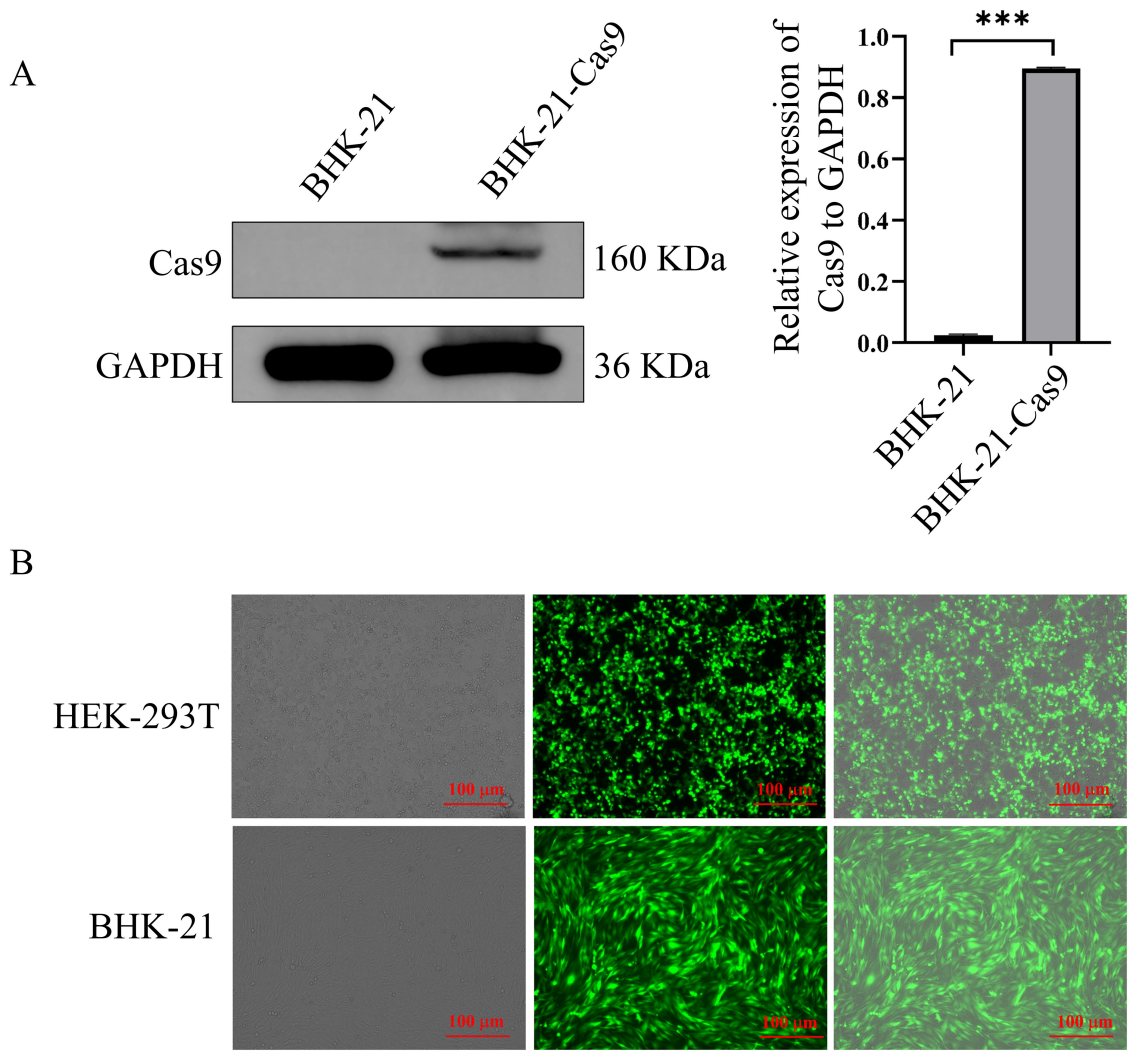
**(A)** Western blot analysis of Cas9 protein expression in BHK-21 cells. **(B)** Analysis of pLenti-GFP lentivirus packaging in 293T cells and transduction efficiency in BHK-21 cells. The symbol "***" indicates a statistically significant difference with *P* < 0.001.

To enable genome-wide screening in BHK-21 cells, a stable BHK-21 cell line expressing Cas9 was first established via lentiviral transduction (**Figure 1A**). HEK293T cells were co-transfected with the library plasmid and control plasmid pLenti-GFP together with helper packaging plasmids, and lentiviral supernatants were collected 48 h later to transduce BHK-21 cells. Robust green fluorescence was observed in cells transduced with the pLenti-GFP control lentivirus, indicating efficient lentivirus production and transduction (**Figure 1B**).

Lentiviral titers of the GeCKO library were determined by quantifying the number of surviving cells following transduction at different infection gradients. The calculated MOIs were 0.88, 0.33, 0.093, 0.034, and 0.023. At an MOI of 0.3, the volume of lentivirus required to infect 1 × 10^5^ BHK-21-Cas9 cells was 80.67 µL (**Table 2**). Collectively, these results confirm that library amplification, lentivirus production, and transduction conditions were suitable and met the requirements for subsequent genome-wide CRISPR/Cas9 screening experiments.

**Table 2:** GeCKO library lentivirus titer assay.

Viral infection volume (μL)	Number of surviving cells (cells/mL)	
200	1.05 × 10^6^	1.12 × 10^6^
100	4.08 × 10^5^	4.15 × 10^5^
50	1.05 × 10^5^	1.25 × 10^5^
25	4.01 × 10^4^	4.25 × 10^4^
12.5	2.75 × 10^4^	3.02 × 10^4^
0	1.21 × 10^6^	1.25 × 10^6^

### 3.2 Genome-wide CRISPR/Cas9 screening identifies host regulatory factors in EHV-1 infection

**Figure 2:**
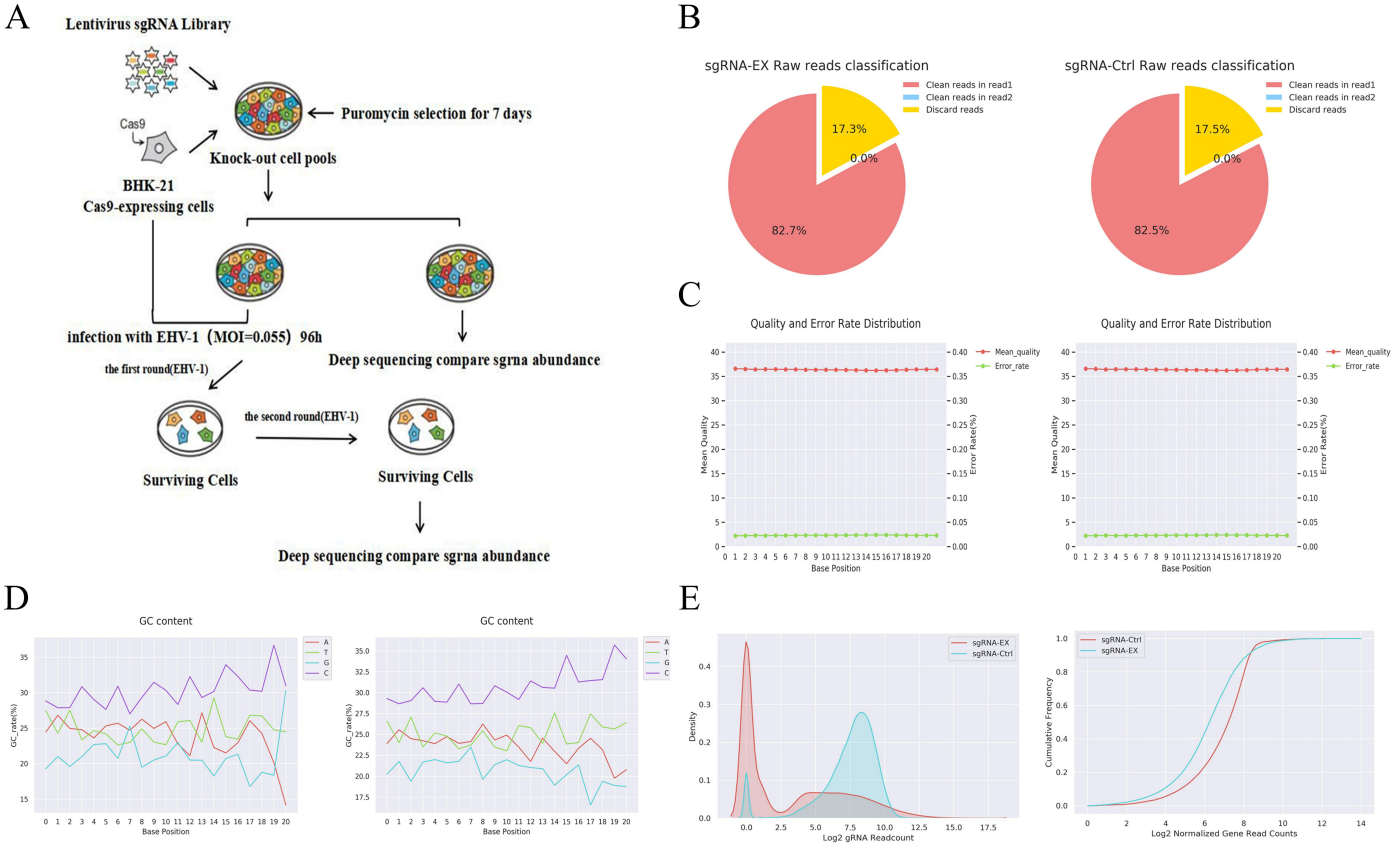
**(A)** Schematic of GeCKO library screening. **(B)** Number of gRNA sequences retrieved from the sgRNA-EX and sgRNA-Ctrl groups in the GeCKOD raw reads classification and their proportion in the raw data. **(C)** Base position plot of reads; the vertical axis represents the average quality score at the corresponding base position; left: sgRNA-EX group; right: sgRNA-Ctrl group. **(D)***X*-axis: base position in the read sequence; *Y*-axis: percentage of the corresponding base type in total bases. **(E)***X*-axis: log_2_ value of read count for a single gRNA on the genome; *Y*-axis: distribution density, i.e., the probability distribution of each gRNA read count. **(E)** Log_2_ value of normalized read counts; *Y*-axis: cumulative frequency. A steeper slope indicates a more uniform distribution of reads across genes in the sample. The baseline group typically exhibits a steeper slope than the control and treatment groups.

To evaluate the robustness and overall quality of the genome-wide CRISPR/Cas9 screen, key screening parameters were first assessed, including MOI, selection pressure, library coverage, and sgRNA depletion (**Figure 2A**). The GeCKO library was introduced into BHK-21-Cas9 cells at a low MOI of 0.3 to ensure predominantly single sgRNA integration per cell, and effective puromycin selection eliminated all wild-type cells, confirming adequate screening pressure.

High-throughput sequencing of both control and virus-selected libraries demonstrated high data quality, with clean read ratios exceeding 82% and a low overall base error rate of 0.02% (**Figures 2B–D**; **Table 3**). Following alignment to the reference sgRNA library, the control group retained high library coverage (95.21%), whereas the virus-selected group exhibited a substantial reduction in coverage (55.74%), consistent with strong negative selection imposed by repeated EHV-1 infection. Kernel density and cumulative distribution analyses further revealed a global shift toward lower sgRNA read counts in the virus-selected group compared with controls (**Figure 2E**).

**Table 3:** Statistics of sequencing data.

Sample ID	sgRNA-EX	sgRNA-Ctrl
Raw reads	54,462,737	61,225,771
Raw bases (G)	16.34	18.37
Clean reads	45,028,514	50,499,854
Clean bases (G)	0.90	1.01
Effective rate (%)	82.68	82.48
Q20 (%)	98.76	98.79
Q30 (%)	96.19	96.24
Error rate (%)	0.02	0.02
GC ratio (%)	50.82	51.20

Notably, 338 sgRNAs were undetected in the virus-selected group, whereas only four sgRNAs were lost in the control group, indicating pronounced sgRNA depletion driven by viral challenge (**Table 4**). Collectively, these findings demonstrate that the CRISPR/Cas9 screen was technically robust and generated a reliable dataset for the identification of host factors involved in EHV-1 infection.

**Table 4:** Statistical table of sample comparison information.

Sample ID	sgRNA-EX	sgRNA-Ctrl
Read	45,028,514	50,499,854
Mappedreads	34,913,440 (77.54%)	39,961,344 (79.13%)
Grna_mean_depth	497	333
Max_depth	203,268	14,128
Median_depth	1	234
Totalgrnas	125,793	125,793
Zerogrnas	55,672	6,024
Coverage_rate	55.74%	95.21%
Totalgenes	22,512	22,512
Zerogenes	338	4

### 3.3 Analysis of principal host factors regulating EHV-1 replication

**Figure 3:**
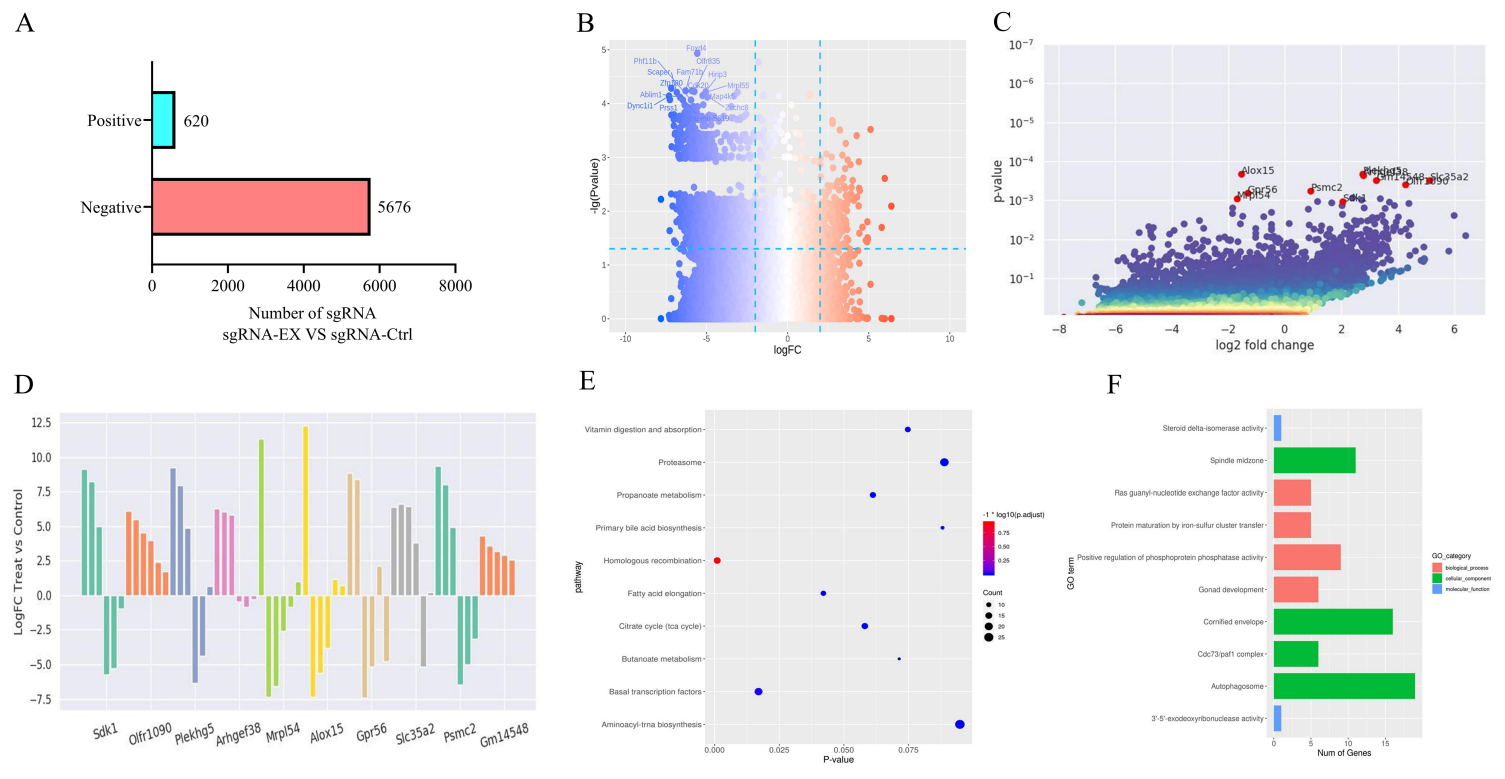
**(A)** Distribution of screening hits: 5,767 negatively selected genes and 620 positively selected genes. **(B)** Volcano plot of screening results: negatively selected genes are shown in blue, positively selected genes in red, and genes without significant selection signals in gray. **(C)** Top 10 most significant genes from the positive selection screen. **(D)** Log_2_ fold-change values of gRNAs targeting the top 10 positively selected genes. **(E)** Bubble chart of KEGG pathway enrichment analysis performed on differentially selected genes. **(F)** Bar graph of Gene Ontology (GO) functional enrichment analysis of differentially selected genes.

Genome-wide CRISPR/Cas9 screening identifies host factors through both positively and negatively selected genes. Genes with positive log_2_ fold-change values are considered positively selected, indicating that their knockout suppresses viral replication, whereas genes with negative log_2_ fold-change values are negatively selected. Using this approach, a total of 5,767 negatively selected genes, including *Foxd4*, *Card14*, and *Cdk20*, and 620 positively selected genes, including *Plekhg5*, *Alox15*, and *Slc35a2*, were identified (**Figure 3A**).

Differential gene enrichment between the experimental and control groups was analyzed using the robust rank aggregation algorithm implemented in MAGeCK software, followed by normalization and statistical testing (**Figure 3B**). Based on sgRNA-level and gene-level counts, the 10 most significantly enriched genes were identified and ranked (**Table 5**; **Figures 3C, D**).

**Table 5:** Statistical table of positive screening results.

Gene ID	Neg score	Neg *P*-value	Neg fdr	Neg rank	Neg lfc	Neg goodsgrna
Plekhg5	0.0000243	0.00020653	1	1	2.7538	4
Alox15	0.000025	0.00020697	1	2	−1.5534	3
Slc35a2	0.0000327	0.00030286	1	3	5. 1118	5
Olfr1090	0.000041	0.00039436	1	4	4.2567	6
Psmc2	0.0000556	0.00058131	1	5	0.91244	3
Arhgef38	0.0000559	0.00023292	1	6	2.7667	3
Gpr56	0.00006	0.00063454	1	7	−1.323	3
Gm14548	0.0000655	0.0003099	1	8	3.2215	5
Mrpl54	0.0000751	0.00091387	1	9	−1.7033	2
Sdk1	0.0000905	0.0010498	1	10	2.0343	3

### 3.4 KEGG and GO functional enrichment analyses of differentially expressed genes

Within organisms, different genes coordinate to perform biological functions. The most critical biochemical metabolic pathways and signal transduction pathways involving high-frequency mutation genes can be identified through pathway significance enrichment analysis.

KEGG enrichment analysis was performed for pathways associated with the top 10 genes ranked by differential screening results (positive rank and negative rank). These genes were mainly enriched in vitamin digestion and absorption, proteasome, propionic acid metabolism, primary bile acid biosynthesis, homologous recombination, fatty acid elongation, the citric acid cycle (TCA cycle), butyric acid metabolism, basal transcription factors, and aminoacyl-tRNA biosynthesis, as well as other metabolic and signaling pathways (**Figure 3E**).

GO classification analysis was conducted for the top 10 genes (positive rank and negative rank) based on differential screening results. In terms of biological processes, synaptic vesicle cycle regulation, apoptosis, regulation of bone marrow cell differentiation, positive regulation of mitochondrial cytochrome c release, elastic fiber-related molecular functions, negative regulation of glial cell migration, pericyte differentiation, regulation of membrane invagination, and chromosome condensation were significantly enriched in functional modules (**Figure 3F**).

### 3.5 Functional gene knockout affects EHV-1 expression levels

**Figure 4:**
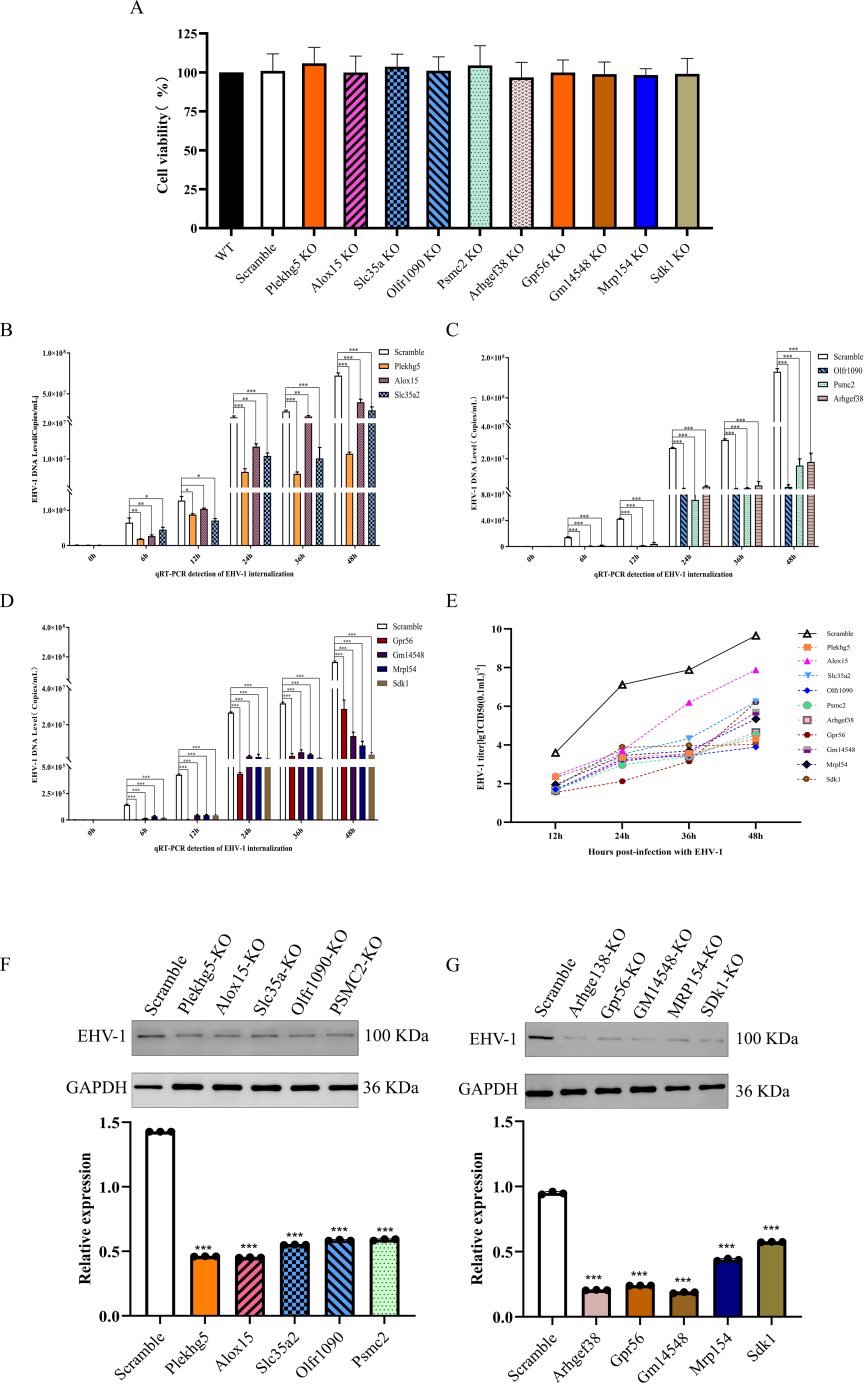
**(A)** Cell viability was assessed using the CCK-8 assay. **(B–D)** Real-time quantitative PCR for detecting EHV-1 viral DNA copy numbers. Data are presented as mean ± SD from three independent biological replicates (*n* = 3). **(E)** Viral titer measurements at different time points following EHV-1 infection (*n* = 5). **(F, G)** Western blot analysis for detecting EHV-1-associated protein expression levels. The symbol "***" indicates a statistically significant difference with *P* < 0.001.

To validate the roles of candidate genes identified by CRISPR screening in EHV-1 infection, knockout cell lines were generated for the top 10 positively selected genes. Intracellular EHV-1 DNA replication levels were then quantified by quantitative PCR at different time points following infection. At 6 h post-infection, EHV-1 DNA levels were significantly reduced in cells with knockout of *Plekhg5*, *Alox15*, *Slc35a2*, *Olfr1090*, *Psmc2*, *Arhgef38*, *Gpr56*, *Gm14548*, *Mrpl54*, and *Sdk1*, indicating that disruption of these genes suppresses EHV-1 replication. Importantly, CCK-8 assays confirmed that knockout of these genes did not significantly affect cell viability, excluding cytotoxic effects as a cause of the reduced viral replication (**Figures 4A–D**).

To further confirm the effects of these functional genes on viral replication, viral titers were determined using the Reed–Muench method at multiple time points post-infection. Compared with the scramble control group, EHV-1 titers were significantly reduced in all corresponding knockout cell lines, further demonstrating that loss of these genes impairs efficient EHV-1 replication (**Figure 4E**).

In addition, EHV-1 protein expression was assessed by Western blot analysis at 48 h post-infection. Protein levels of EHV-1 were markedly decreased in all functional gene knockout cells compared with those in the scramble control group, indicating that deletion of these genes not only reduces viral DNA replication but also inhibits viral protein synthesis (**Figures 4F, G**). Collectively, these findings demonstrate that knockout of positively selected host genes significantly suppresses EHV-1 DNA replication, virus production, and protein expression, highlighting their critical roles in the EHV-1 replication cycle.

### 3.6 Gene knockout alleviates CPEs induced by EHV-1 infection

**Figure 5:**
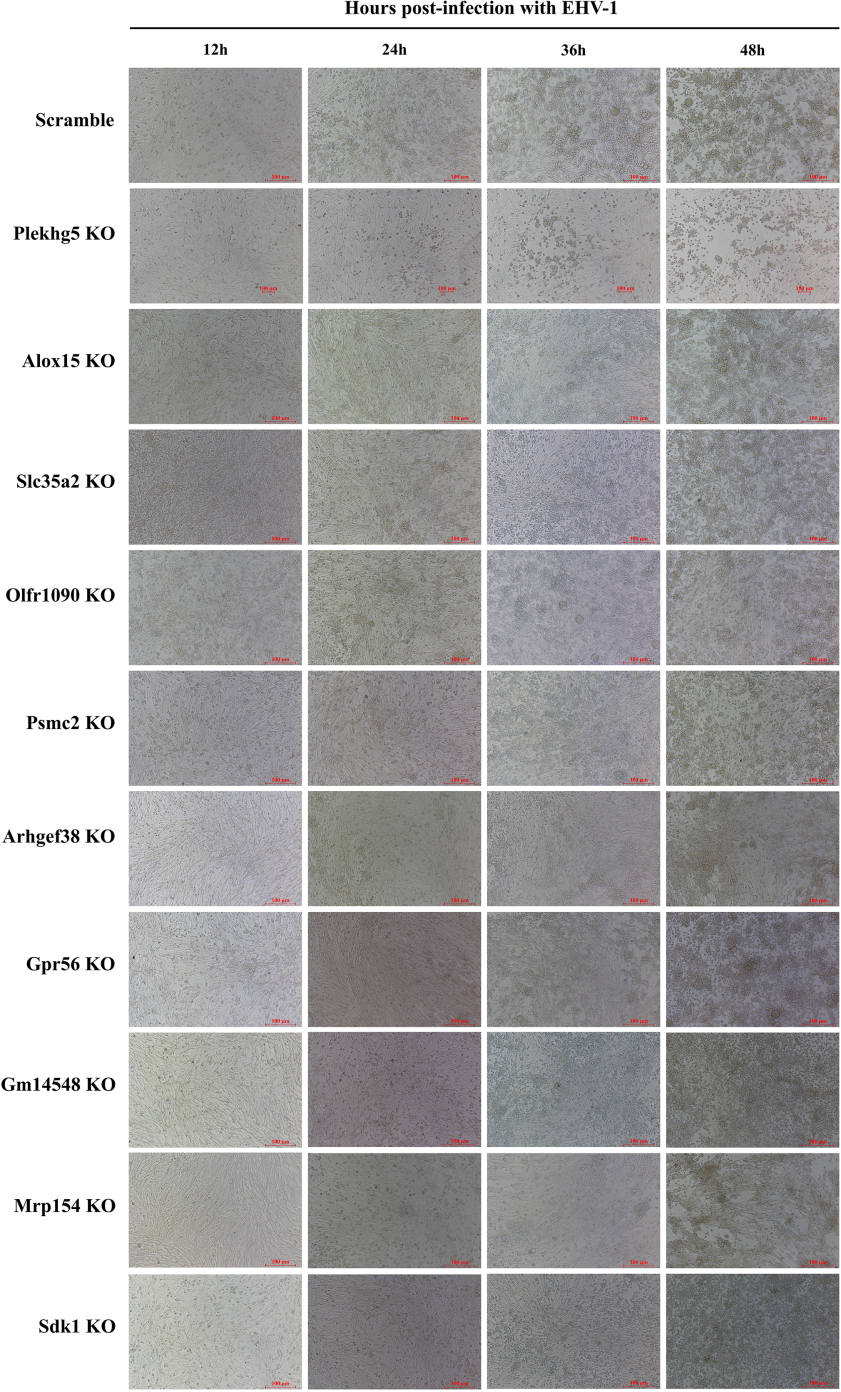
Observation of cytopathic effects in gene knockout cells following EHV-1 infection.

To further examine the impact of functional gene knockout on CPEs following EHV-1 infection, viral pathogenicity was compared between scramble control cells and gene knockout cells. In scramble control cells, EHV-1 infection for 24, 36, and 48 h resulted in pronounced CPEs, characterized by cell rounding, contraction, and detachment. In contrast, at the same time points, cells with knockout of the functional genes exhibited delayed onset of cytopathic changes, better preservation of cellular morphology, and markedly reduced CPE severity. These findings indicate that knockout of the identified functional genes effectively delays EHV-1-induced cytopathic progression and partially attenuates viral cytotoxicity (**Figure 5**).

## 4 Discussion

Host factors play indispensable roles throughout the viral life cycle, influencing viral entry, replication, assembly, and release (18). Systematic identification of these host determinants through genome-wide CRISPR/Cas9 screening has become a powerful strategy for delineating virus–host interaction networks (19, 20). Although this approach has been widely applied to several human herpesviruses, including HSV-1 and human cytomegalovirus, comprehensive analyses of host-dependent factors involved in EHV-1 replication remain limited. Accordingly, this study employed a genome-wide CRISPR/Cas9 screen to identify host genes required for efficient EHV-1 replication, thereby expanding current understanding of EHV-1–host interactions and providing new insights into potential antiviral targets.

Stable Cas9 expression in host cells is a prerequisite for successful CRISPR-based screening (9, 21). In this study, BHK-21 cells, which are highly susceptible to EHV-1 and widely used in EHV-1 research, were selected to generate and validate monoclonal cell lines stably expressing Cas9 (BHK-21-Cas9) via lentiviral transduction. On this basis, the electroporation efficiency of the GeCKO library and the quality of lentiviral packaging were rigorously evaluated. The achieved transformation efficiency (3.26 × 10^8^) and library coverage satisfied the requirements for robust library amplification, which is essential for ensuring the reliability and reproducibility of screening outcomes. Subsequently, a pooled GeCKO library lentivirus was produced and titrated across multiple infection gradients, revealing that a lentiviral volume of approximately 80.67 µL was required to achieve an MOI of 0.3. This parameter provided a solid foundation for subsequent large-scale cell transduction and screening. Using the GeCKO library, a genome-wide CRISPR/Cas9 functional screen was then performed under EHV-1 infection conditions to systematically delineate the host gene networks governing EHV-1 infection.

After rigorous verification of library amplification, lentiviral packaging, and infection conditions, BHK-21-Cas9 cells were subjected to multiple rounds of EHV-1 challenge. Sequencing analysis revealed that, under EHV-1-mediated selective pressure, sgRNA library coverage in the experimental group (sgRNA-EX) was markedly reduced compared with the untreated control group. Specifically, 338 sgRNAs were undetectable in the experimental group, whereas only four sgRNAs were lost in the control group. This pronounced sgRNA depletion represents a typical negative-selection phenotype, indicating that the corresponding genes are essential for cell survival under viral infection. The progressive reduction in library coverage, the shift of the density distribution toward low-abundance sgRNAs, and the overall high quality of sequencing data collectively demonstrate that repeated EHV-1 infection imposed strong selective pressure on host genes, consistent with previous CRISPR-based screening studies (22). Robust rank aggregation analysis implemented in MAGeCK identified 5,767 negatively selected genes and 620 positively selected genes, highlighting the extensive interaction network between EHV-1 and host determinants. Among negatively selected genes, sgRNA enrichment following viral infection suggests that these genes may function as restrictive host factors limiting viral replication. In contrast, sgRNA depletion among positively selected genes indicates that loss of these genes suppresses viral replication, implying that they act as host-dependent factors that facilitate EHV-1 infection.

Notably, as a member of the subfamily Alphaherpesvirinae, EHV-1 replication is highly dependent on the coordinated regulation of diverse host cellular processes, including metabolic reprogramming, membrane system remodeling, vesicular transport, cell-cycle control, and immune evasion (2, 23, 24). Consistent with this complexity, both positive and negative host regulators with distinct cellular functions were identified in our screen. These findings align with established herpesvirus–host interaction paradigms and further highlight the extensive exploitation and modulation of host cellular networks during EHV-1 infection.

The Kyoto Encyclopedia of Genes and Genomes pathway enrichment analysis revealed that differentially selected genes were enriched in pathways associated with proteasome function, mitochondrial metabolism, fatty acid and bile acid metabolism, transcriptional regulation, and aminoacyl-tRNA biosynthesis. These pathways are fundamental to cellular energy production, protein homeostasis, and macromolecule synthesis—processes that herpesviruses are known to hijack to support viral DNA replication and protein expression. Gene Ontology enrichment analysis further highlighted biological processes including vesicle-mediated transport, regulation of apoptosis, mitochondrial function, and chromatin organization. Collectively, these pathways and processes are closely linked to cellular energy metabolism, protein homeostasis, macromolecular biosynthesis, and membrane trafficking, all of which are recognized as host functions exploited by herpesviruses to facilitate productive replication. These findings suggest that EHV-1 replication relies on coordinated regulation of host metabolic and structural networks.

From the positively selected genes, 10 candidates (*Plekhg5*, *Alox15*, *Slc35a2*, *Olfr1090*, *Psmc2*, *Arhgef38*, *Gpr56*, *Gm14548*, *Mrpl54*, and *Sdk1*) were prioritized for functional validation. Using CRISPR/Cas9, corresponding gene knockdown BHK-21 cell lines were generated to assess the impact of host gene disruption on EHV-1 replication. The results demonstrated that knockout of each candidate gene significantly suppressed EHV-1 DNA replication at 12 h post-infection. This inhibitory effect was further supported by marked reductions in viral titers and viral protein expression, indicating that loss of these genes attenuates EHV-1 replication at multiple stages, including genome replication, transcription, and protein synthesis. Consistent with these findings, scramble control cells exhibited extensive CPEs by 48 h post-infection, characterized by cell rounding, contraction, and widespread detachment. In contrast, cells lacking the functional genes maintained substantially more intact morphology at the same time point, with clearly alleviated CPEs. Importantly, cell viability assays confirmed that, in the absence of infection, knockout of these genes did not significantly impair basal cell growth, indicating that the observed antiviral effects were not attributable to non-specific cytotoxicity.

Several validated host factors are involved in conserved cellular processes previously implicated in herpesvirus biology. For instance, *Slc35a2* encodes a nucleotide-sugar transporter essential for protein glycosylation, a process critical for the maturation of viral envelope glycoproteins (25). *Psmc2* is a core component of the proteasome, and this complex has been implicated in regulating herpesviral protein turnover and viral genome replication (26, 27). Other candidates, such as *Plekhg5* and its family members, connect the cytoskeleton to viral replication, supporting the notion that EHV-1 exploits host intracellular trafficking (28–30). Notably, several genes identified in this study have not previously been linked to herpesvirus infection, suggesting the existence of EHV-1-specific host dependencies that warrant further investigation.

Several limitations of this study should be acknowledged. First, the CRISPR library used was designed based on the mouse genome, whereas screening was performed in hamster-derived BHK-21 cells. Although target sequence conservation was verified, interspecies differences may still influence knockout efficiency for certain genes. Second, functional validation was limited to BHK-21 cells; future studies should confirm physiological relevance using equine-derived cell lines or primary equine cells. Third, rescue experiments, such as gene complementation, were not performed; therefore, gene-specific effects cannot yet be fully established. Finally, although targeting host factors represents an attractive strategy for reducing the likelihood of antiviral resistance, interventions directed at essential cellular pathways require careful evaluation of safety profiles and therapeutic windows.

In conclusion, this study represents the first genome-wide CRISPR/Cas9 screen to systematically identify host factors essential for EHV-1 replication. The findings provide new insights into the cellular pathways exploited by EHV-1 and establish a foundation for future mechanistic investigations and the development of host-targeted antiviral strategies. Nevertheless, further validation in equine-relevant models and detailed mechanistic studies will be necessary to fully assess the translational potential of these candidate host factors.

## Data Availability

The datasets presented in this study can be found in online repositories. The names of the repository/repositories and accession number(s) can be found below: The data presented in the study are deposited in the Sequence Read Archive (SRA) repository, ac-cession number PRJNA1330729.

## Supplementary material

Supplementary Figure 1 Plekhg5, Alox15, Slc35a2, Olfr1090, Psmc2, Arhgef38, Gpr56, Gm14548, Mrpl54, and Sdk1 mRNA-level qPCR validation in knockout cells.
