# Correction: Genome-wide CRISPR/Cas9 screening identifies host factors critical for antiviral defense against equine herpesvirus type 1

**DOI:** 10.3389/fimmu.2026.1817672

**Published:** 2026-03-12

**Authors:** Ziqian Li, Lijuan Ge, Tingting Yu, Shubing Lv, Qiang Fu, Huijun Shi

**Affiliations:** 1 College of Veterinary Medicine, Xinjiang Agricultural University, Urumqi, Xinjiang Uygur Autonomous Region, China; 2 Key Laboratory of New Drug Research and Innovation for Herbivorous Animals of Xinjiang, Urumqi, Xinjiang Uygur Autonomous Region, China

**Keywords:** CRISPR/Cas9, EHV-1, library screening, viral host factors, viral replication

The order of authors in the author list of the published paper was erroneously given as:

”Ziqian Li^1^, Tingting Yu^1^, Lijuan Ge^1^, Shubing Lv^1^, Qiang Fu^1,2*^, Huijun Shi^1,2*^”

The correct author list reads:

“Ziqian Li^1^, Lijuan Ge^1^, Tingting Yu^1^, Shubing Lv^1^, Qiang Fu^1,2*^, Huijun Shi^1,2*^”

The original version of this article has been updated.

Also, there was a mistake in **Table 2**: GeCKO library lentivirus titer assay as published. In the column header “Number of surviving cells (cells/mL)”, the numerical values listed under this header are actually intended to be expressed as multiples of 10^6^ (i.e., ×10^6^ cells/mL). However, during the typesetting process, the superscript “6” was not formatted correctly, resulting in the values being displayed as plain numbers (e.g., “10^6^” instead of “×10^6^”). The corrected **Table 2**: GeCKO library lentivirus titer assay appears below.

**Table 2:** GeCKO library lentivirus titer assay.

Viral infection volume (μL)	Number of surviving cells (cells/mL)	
200	1.05 × 10^6^	1.12 × 10^6^
100	4.08 × 10^5^	4.15 × 10^5^
50	1.05 × 10^5^	1.25 × 10^5^
25	4.01 × 10^4^	4.25 × 10^4^
12.5	2.75 × 10^4^	3.02 × 10^4^
0	1.21 × 10^6^	1.25 × 10^6^

The original version of this article has been updated.

