# Supplementary figures and images for: Genome-wide CRISPR/Cas9 screening identifies host factors critical for antiviral defense against equine herpesvirus type 1
Source: Front Immunol. 2026 Feb 2;17:1764863. doi: 10.3389/fimmu.2026.1764863 (PMC12907326; doi:10.3389/fimmu.2026.1764863)

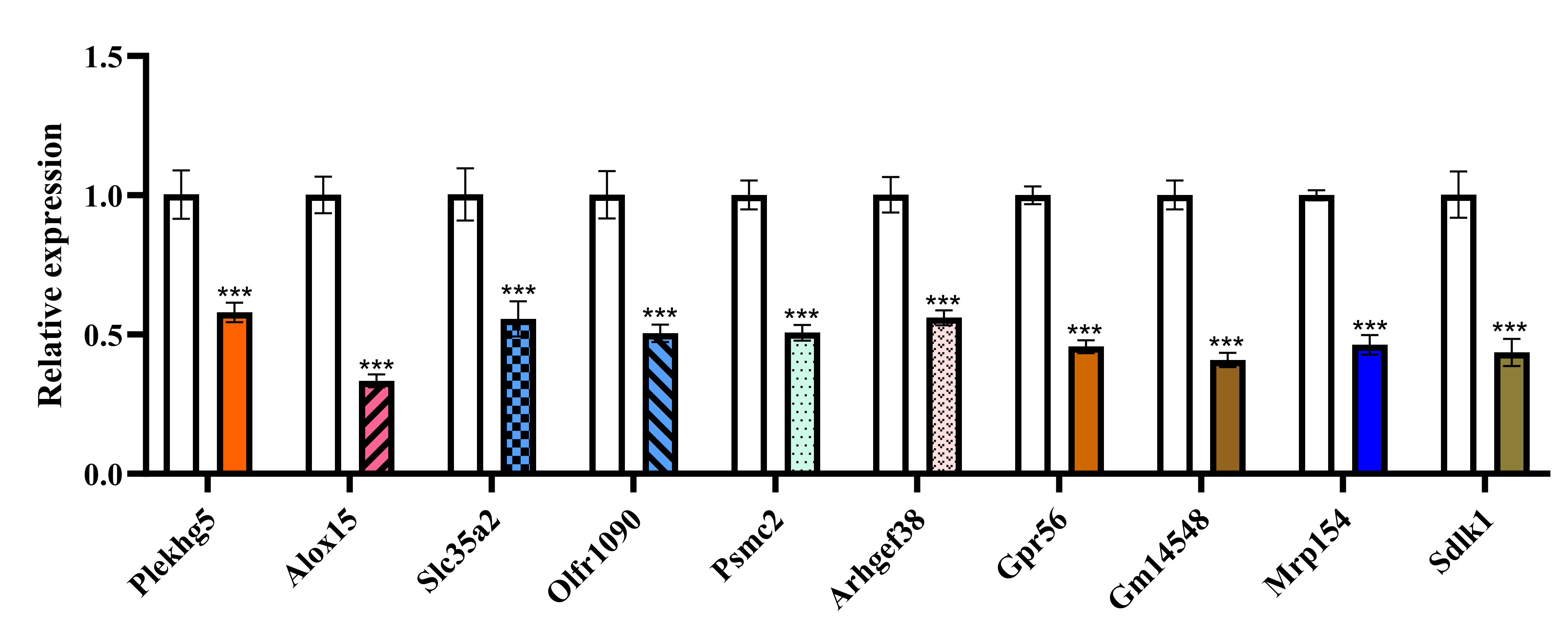

Supplement: Supplementary Figure 1 — Plekhg5, Alox15, Slc35a2, Olfr1090, Psmc2, Arhgef38, Gpr56, Gm14548, Mrpl54, and Sdk1 mRNA-level qPCR validation in knockout cells. [file Image1.tif]

Figure 1A

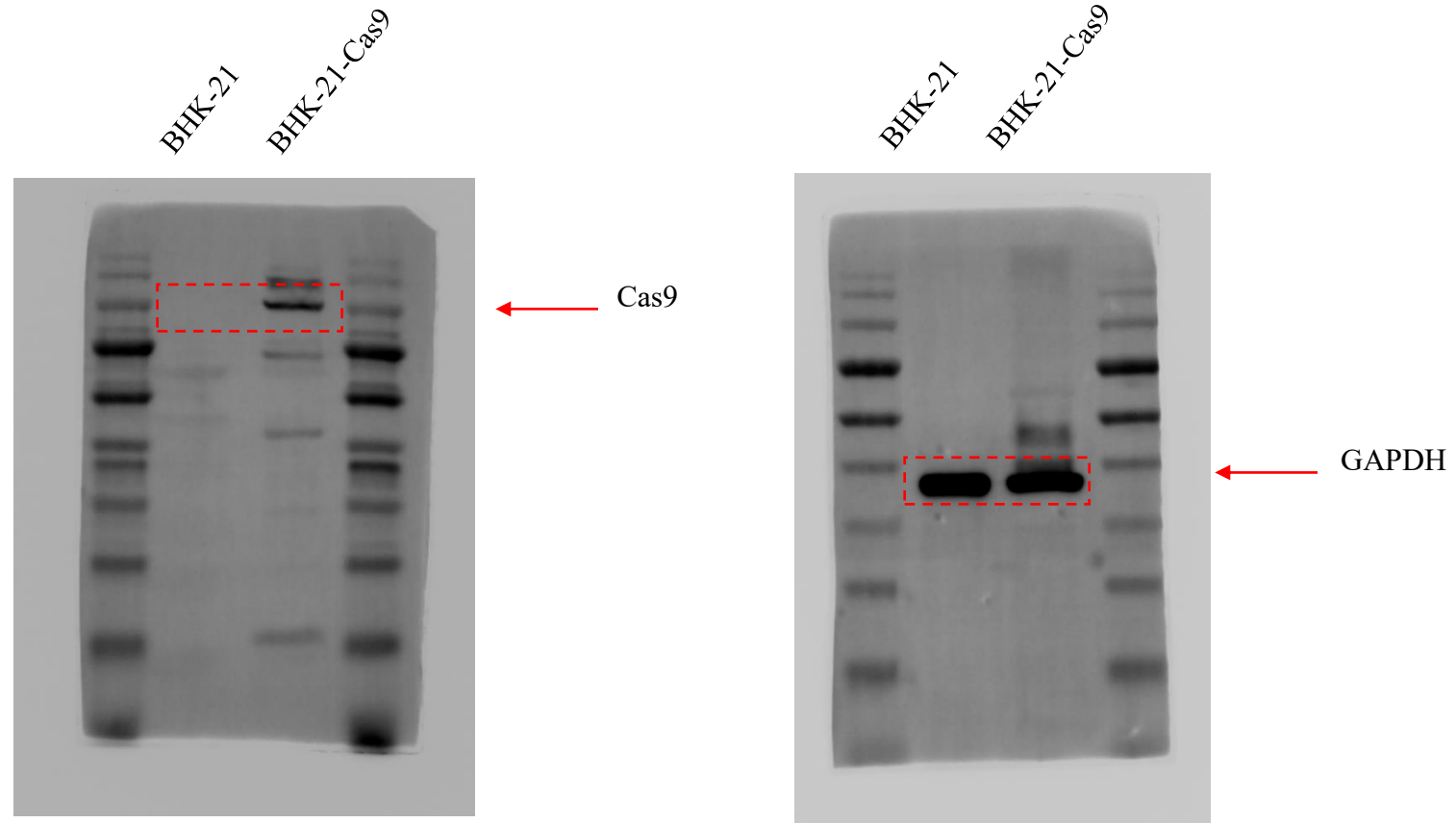

Supplement: Supplementary file 2 [file DataSheet1.pdf]

Figure 4E

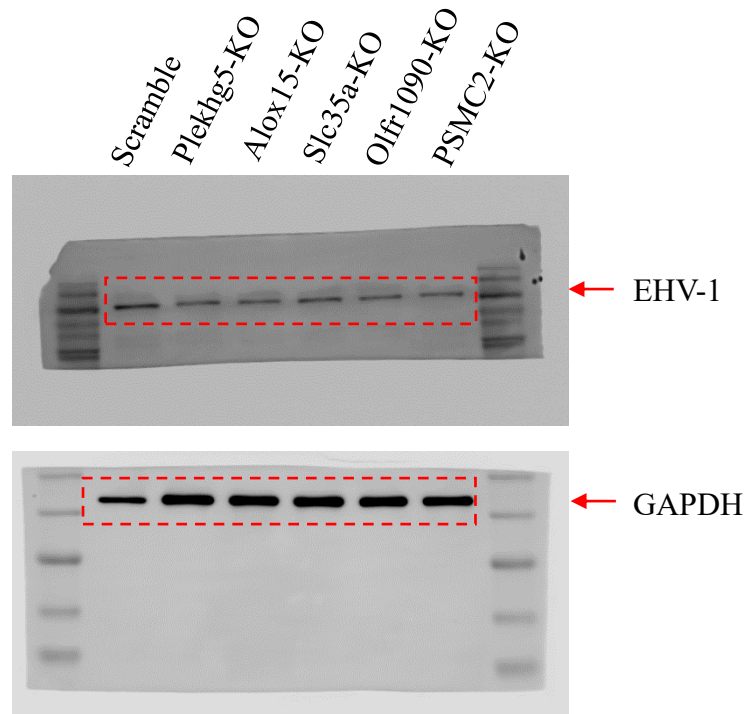

Figure 4F

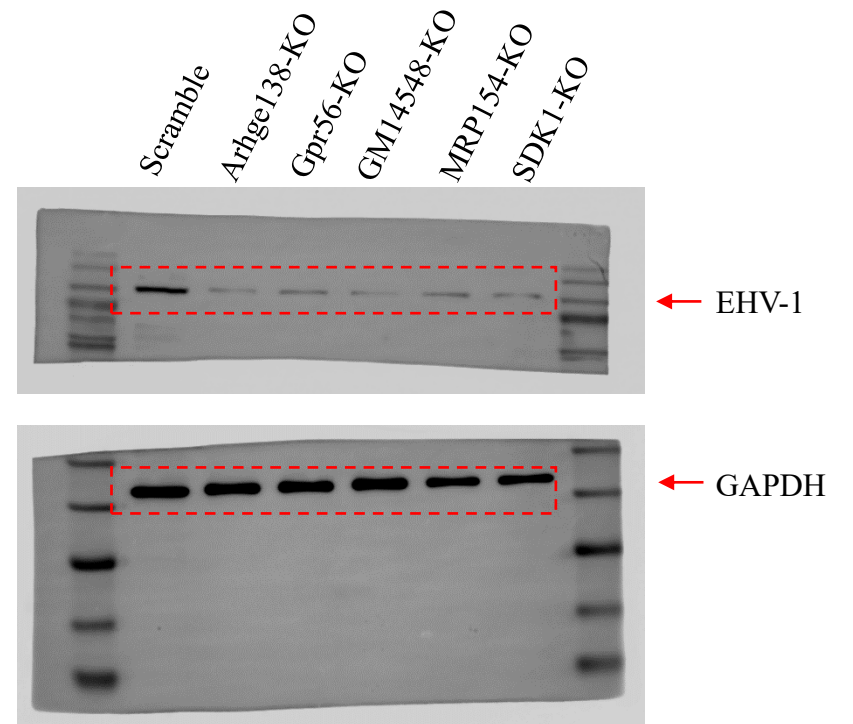

Supplement: Supplementary file 3 [file DataSheet2.pdf]
